# Editorial: Nutrigenomics and personalized nutrition: advancing basic, clinical, and translational research

**DOI:** 10.3389/fnut.2024.1435475

**Published:** 2024-06-14

**Authors:** George Lagoumintzis, Nikolaos A. Afratis, George P. Patrinos

**Affiliations:** ^1^Department of Pharmacy, School of Health Sciences, University of Patras, Patras, Greece; ^2^Department of Agriculture Development, Agri-Food and Natural Resources Management, National and Kapodistrian University of Athens, Psachna, Greece; ^3^Department of Immunology and Regenerative Biology, Weizmann Institute of Science, Rehovot, Israel; ^4^Department of Genetics and Genomics, College of Medicine and Health Sciences, United Arab Emirates University, Al-Ain, United Arab Emirates; ^5^Zayed Center for Health Sciences, United Arab Emirates University, Al-Ain, United Arab Emirates; ^6^Department of Pathology, Clinical Bioinformatics Unit, Faculty of Medicine and Health Sciences, Erasmus University Medical Center, Rotterdam, Netherlands

**Keywords:** personalized nutrition, dietary advice, genetic makeup, nutritional intervention, genetic variation, nutrigenetics/nutrigenomics, metabolism, healthcare

Personalized nutrition, also known as nutrigenomics, focuses on providing genome-guided, customized dietary advice and interventions. This approach, which considers an individual's specific nutritional needs, genetic makeup, health status, lifestyle, and personal preferences (1), is gaining importance with advancements in our understanding of genetics, metabolism, and nutrition (2, 3). Developing unbiased, customized dietary recommendations requires consideration of a diverse array of factors, including nutrigenomics and deep phenotyping (4). This Research Topic aimed to gather, evaluate, and publish cutting-edge submissions in the field of personalized nutrition. Additionally, we seek to analyze key factors influencing an individual's response to lifestyle and nutritional interventions, encompassing genomic to phenotypic variations.

Genetic variations influence food metabolism and individual responses to dietary intake, which is crucial for improving health and preventing disease. The complex interactions between genes and nutrients, influenced by various genetic and environmental factors, necessitate a deeper understanding. A genome-wide association study (GWAS) by Hendi et al. explored the genetic architecture contributing to Vitamin D deficiency in the Qatari population. Using whole-genome sequencing data from 6,047 subjects in the Qatar Biobank project, the researchers identified genetic determinants of Vitamin D levels in Middle Easterners, revealing consistent patterns in the effect size and allele frequency of common variants. Notably, a primary genetic determinant of Vitamin D predisposition in Middle Eastern individuals was identified as a polymorphism in the GC gene.

The causal roles of certain foods, nutrients, and other nutritional factors in health and disease are only partially established, as most information comes from standard observational studies. These studies often include dietary intake misclassification, residual confounding from correlated factors, and reverse causation bias. Mendelian randomization (MR) strengthens causal inference about modifiable exposures and disease risk using germline genetic variation. MR is less prone to confounding, reverse causality, and measurement error than conventional observational approaches, though it has its own biases. Li et al. used a two-sample MR analysis to address the causal associations between essential nutrients (amino acids, polyunsaturated fatty acids, minerals, and vitamins) and cerebral small vessel disease (CSVD). They identified gene-environment interactions indicating that essential nutrients impact the risk of CSVD, providing insights that could inform nutritional intervention strategies.

Modern metabolism encompasses not only the chemical conversion of food into energy and other byproducts but also the impact of food as a conditioning environment that influences genome function and physiology. Hellbach et al. conducted an epigenome-wide association study to investigate the intricate relationship between usual dietary intake and alterations in DNA methylation patterns in blood mononuclear cells. Their findings unveiled striking relationships between food intake and changes in DNA methylation, particularly with the consumption of cream and spirits. These changes were annotated in CLN3, PROM1, DLEU7, TLL2, and UGT1A10 genes. These results may have profound implications for personalized nutrition, suggesting that specific food items can significantly affect DNA methylation patterns. However, the weak associations with other food ingredients indicate that larger cohorts may be necessary to delineate these relationships further.

Considering the impact of dietary factors and how their metabolites influence and control metabolic reactions, it is widely accepted that excessive calorie consumption, particularly from saturated and trans fats, is closely linked to obesity and other diseases prevalent in Western societies. Sandoval et al. systematically reviewed the role of long-chain polyunsaturated fatty acids, specifically eicosapentaenoic acid (EPA) and docosahexaenoic acid (DHA), in modulating gene expression during obesity progression. Their work suggests that the incorporation of EPA and DHA may offer potential benefits in addressing non-communicable diseases, including obesity, due to their anti-inflammatory properties and their ability to regulate genes associated with obesity, such as PPARγ and ALOX.

In today's omics era, integrating Artificial Intelligence (AI) and Machine Learning (ML) technologies into nutrigenomics and nutrigenetics unlocks new potentials for personalized nutrition, offering more precise and efficient ways to understand the complex interactions between genes, nutrients, and health outcomes. In this perspective, Pigsborg et al. utilized ML to build a predictive model of metabolic markers for successful weight loss in subjects with overweight or obesity undergoing a 6-month dietary intervention with the New Nordic Diet (NND). By combining clinical baseline data with untargeted metabolomics, they identified a model containing two metabolites (adipic acid and argininic acid) that could predict the likelihood of achieving clinically significant weight loss on an *ad libitum* NND. This demonstrates that models based on an untargeted multi-platform metabolomics approach can optimize precision dietary treatment for obesity. In a similar study, Ford et al. explored bioinformatics and digital applications, proposing that healthy dietary approaches can serve as a low-cost, protective, and complementary choice to various pharmaceutical therapies. Their approach, termed “Dietary Rational Gene Targeting” (DRGT), is a therapeutic dietary strategy that uses healthy ingredients to restore disease-causing gene expression to normal. Ford et al. used DRGT to identify human studies assessing gene expression after ingesting healthy dietary agents, using these data to create an online dietary guide app prototype. This app aims to help patients and healthcare providers prevent various health conditions through dietary interventions.

Nutrigenomics and personalized nutrition represent a promising bridge between genetics and nutritional interventions, aiming to transform healthcare by tailoring dietary advice based on genomic insights. This individualized strategy could prevent chronic diseases, maximize health, and improve wellbeing and longevity, highlighting the need for ongoing research and cross-disciplinary collaboration. As our understanding of genes, diet, and health grows, tailored nutrition could significantly improve public health and usher in a new era of personalized medicine. Furthermore, AI technologies such as ML, deep learning, and other sophisticated bioinformatic tools and applications can revolutionize nutrigenomics and nutrigenetics by enabling more precise, personalized, and proactive approaches to nutrition and health. As these technologies continue to advance, they will play a crucial role in transforming dietary recommendations and improving health outcomes on an individual level.

## Author contributions

GL: Writing – review & editing, Writing – original draft, Supervision, Project administration, Conceptualization. NA: Conceptualization, Writing – review & editing, Project administration. GP: Writing – review & editing, Project administration, Conceptualization.
